# The Mediating Role of General and Cognitive Stress on the Effect of an App-Based Intervention on Productivity Measures in Workers: Randomized Controlled Trial

**DOI:** 10.2196/42317

**Published:** 2023-07-03

**Authors:** Carlota de Miquel, Maria Victoria Moneta, Silvana Weber, Christopher Lorenz, Beatriz Olaya, Josep Maria Haro

**Affiliations:** 1 Research, Innovation and Teaching Unit Parc Sanitari Sant Joan de Déu Sant Boi de Llobregat Spain; 2 Centro de Investigación Biomédica en Red de Salud Mental Madrid Spain; 3 Department of Medicine University of Barcelona Barcelona Spain; 4 Psychology of Communication and New Media Human-Computer-Media Institute University of Würzburg Würzburg Germany; 5 Institut für Mathematik Goethe-Universität Frankfurt Frankfurt Germany

**Keywords:** e-mental health intervention, work, absenteeism, presenteeism, stress, mediation

## Abstract

**Background:**

Loss of productivity is a result of absence from work (absenteeism) or of working with limitations due to illness (presenteeism). Recently, occupational mental health interventions have increasingly been delivered in digital format, as this is thought to be more convenient, flexible, easily accessible, and anonymous. However, the effectiveness of electronic mental health (e-mental health) interventions in the workplace to improve presenteeism and absenteeism remains unknown, and could be potentially mediated by psychological variables such as stress levels.

**Objective:**

The aim of this study was to determine the effectiveness of an e-mental health intervention to reduce absenteeism and presenteeism in employees, as well as to investigate the mediating role of stress in this effect.

**Methods:**

Employees of six companies in two countries participated in a randomized controlled trial (n=210 in the intervention group and n=322 in the waitlist control group). Participants in the intervention group could use the Kelaa Mental Resilience app for 4 weeks. All participants were asked to complete assessments at baseline, during the intervention, postintervention, and at a 2-week follow-up. Absenteeism and presenteeism were assessed by means of the Work Productivity and Activity Impairment Questionnaire: General Health, while general and cognitive stress were assessed through the Copenhagen Psychosocial Questionnaire-Revised Version. Regression and mediation analyses were performed to evaluate the effect of the Kelaa Mental Resilience app on presenteeism and absenteeism.

**Results:**

The intervention did not have a direct effect on presenteeism or absenteeism, neither at postintervention nor at follow-up. Nevertheless, general stress significantly mediated the intervention effect on presenteeism (*P*=.005) but not on absenteeism (*P*=.92), and cognitive stress mediated the effect of the intervention on both presenteeism (*P*<.001) and absenteeism (*P*=.02) right after the intervention. At the 2-week follow-up, the mediating effect of cognitive stress on presenteeism was significant (*P*=.04), although this was not the case for its mediating effect on absenteeism (*P*=.36). Additionally, at the 2-week follow-up, general stress did not mediate the intervention effect on presenteeism (*P*=.25) or on absenteeism (*P*=.72).

**Conclusions:**

While no direct effect of the e-mental health intervention on productivity was found in this study, our findings suggest that stress reduction could mediate the effect of the intervention on presenteeism and absenteeism. As such, e-mental health interventions that address stress in employees might also indirectly reduce presenteeism and absenteeism in these employees. However, due to study limitations such as an overrepresentation of female participants in the sample and a high proportion of attrition, these results should be interpreted with caution. Future research is needed to better understand the mechanisms of interventions on productivity in the workplace.

**Trial Registration:**

ClinicalTrials.gov NCT05924542; https://clinicaltrials.gov/study/NCT05924542

## Introduction

### Background

Mental disorders and mental health–related conditions such as stress contribute to productivity loss, with an estimated cost of €400 billion (~US $440 billion) every year in Europe [1,2]. Productivity loss due to illness is broadly a result of absence from work (ie, absenteeism) or working with limitations due to the illness (ie, presenteeism) [3], which represents a serious problem for both employees and employers [4].

In recent years, occupational mental health interventions [5,6] have increasingly been delivered in a digital form, because electronic mental health (e-mental health) interventions are thought to be more convenient, flexible, easily accessible, and anonymous [7,8]. They also provide an opportunity for the provision of a proactive, preventative approach for employees’ mental health [6,9]. A meta-analysis of the effectiveness of occupational e-mental health interventions showed a moderate positive effect for different mental health conditions, including stress and burnout [10]. However, there was high heterogeneity between studies, with a lack of understanding of which factors were contributing to the variation in effectiveness [10].

Studies about the effectiveness of e-mental health interventions to reduce absenteeism and presenteeism in the workplace are scarce, and the evidence mainly comes from cost-effective analyses. One example is a study of Happy@Work, a web-based guided self-help intervention to reduce depression at work [11]. While the authors identified presenteeism and absenteeism to be respectively responsible for more than 50% and 30% of the costs in a financial evaluation from both the societal perspective and employers’ perspective, the intervention itself was not considered to be cost-effective or cost-saving for the employer [12]. Another study in this area examined the economic impact of a targeted guided self-help program for employees [13]. This program was found to be cost-saving and to produce a return on investment ratio of 1.05 estimated as a comparison between the cost savings produced by the intervention relative to the intervention costs [13]. Finally, a study that examined a mobile-based and internet-based stress management intervention found a significant reduction of presenteeism, although this reduction was not found for absenteeism [14]. Overall, the effectiveness of these interventions to reduce absenteeism and presenteeism might depend on several factors such as well-being [15]. Indeed, stress at the workplace [16], financial stress [17], and anxiety and depression [18] have been shown to be associated with presenteeism and absenteeism. Thus, interventions targeting and improving the mental health of employees are expected to indirectly improve their work functioning.

One of these factors could be the stress level of workers. Lazarus and Folkman [19] defined psychological stress as “a particular relationship between the person and the environment that is appraised by the person as taxing or exceeding his or her resources and endangering his or her well-being.” Psychological stress can in turn be divided into different types of stress, including cognitive stress (ie, the dimension of stress that has an impact on the cognitive ability of an individual). Cognitive stress has been shown to be directly and negatively related to job control [20] as well as to other types of psychological stress. This lines up with the Job Demands-Resources (JDR) model [21,22], in which stress is characterized as a reaction to an imbalance between the demands that a person’s job places on them and their ability to meet those obligations. People undergo a process of mental and physical health degradation when demands outweigh resources, which results in decreased energy and tiredness. If a motivational process takes place, there is an increase in work engagement and positive outcomes such as greater well-being and organizational commitment [23-25]. Accordingly, studies have shown both work-related and personal stress to be associated with higher presenteeism [18,26,27] and absenteeism [28]. However, the association between stress and absenteeism is not always found [14,18].

### Study Objectives and Hypotheses

The Kelaa Mental Resilience app, a science-based health and well-being mobile phone app provided by Soma Analytics (London, United Kingdom), was found to significantly decrease stress (β=–.15, SE=.04; *P*<.001) over time in a large-scale longitudinal randomized controlled trial (RCT) [29]. The app is a digital tool that focuses on the prevention of mental health problems, rather than their treatment, and is theoretically grounded in the JDR model (eg, [21,30]). The intervention was developed specifically for the workplace and offers a combination of diaries, sensor measurements, and guides [31]. The app was designed to implement lifestyle changes for users and aimed at increasing their resources. As outlined above, an increase in job and personal resources can impact health and organizational outcomes in both direct and indirect manners; thus, increasing workers’ resources might enable them not only to deal with job demands more resiliently but also to use the already available job resources more efficiently [32]. In this study, we aimed to determine whether use of the Kelaa Mental Resilience app significantly decreased the levels of presenteeism and absenteeism in employees. Additionally, since mental health has been found to impact productivity, we also aimed to explore the mediating role of stress levels on the intervention effect in presenteeism and absenteeism measures.

First, we hypothesized that after using the app for 4 weeks, participants in the intervention group would report lower levels of presenteeism and absenteeism than participants in the waitlist control group (direct effect). Second, presenteeism and absenteeism scores were expected to be associated with higher levels of stress in all participants, irrespective of group. Finally, we expected a decrease in stress levels to also have an indirect impact on presenteeism and absenteeism levels (indirect effect).

## Methods

### Study Design and Procedures

This study followed a longitudinal RCT experimental design with a 2-week follow-up. The trial started in January 2018 and ended in September 2018. Human resources managers were blind to the participants’ group allocation and participants were blind to the goals and hypotheses of the study; however, they were not blind to their group allocation. While blinding is of course desirable in practice, a naturalistic study is often not possible to achieve in a digital health intervention.

Participants were randomly assigned to one of the two experimental conditions (intervention group or waitlist control group) after signing up and giving their informed consent. Randomization was implemented through a software program invoking an unbiased random number generator; subjects were assigned to a group without human intervention immediately after giving their consent. Measurements of all participants were collected online via Qualtrics at baseline (T0, week 0), midintervention (T1, week 2), postintervention (T2, week 4), and at the 2-week follow-up (T3, week 6). Participants belonging to the app (intervention) group were asked to first complete the questionnaires before downloading and starting to use the app. Reminders and invitations to the follow-up questionnaires were sent via email. Participants had 7 days to complete the questionnaires. As soon as they had completed the follow-up measurement, all participants were thanked and fully debriefed.

The intervention lasted 4 weeks. During these 4 weeks, participants in the intervention group could complete a maximum of 28 sessions (1 per day) and track a maximum of 28 nights. However, it was completely up to the user to decide to what extent they wanted to engage with the app. They could also decide whether to use the app on their personal phone or their work phone. Reminders were sent through push notifications, although users had the option to turn them off. After the 4 weeks of the intervention, the interventional module within the app was withdrawn for the intervention group. A 2-week follow-up measurement was then taken to assess which gains from the 4-week intervention persisted. Participants belonging to the waitlist control group had no access to the intervention for the duration of the trial (ie, 6 weeks). They received access to the intervention and the tracking modules once the study was completed.

### Participants

**Figure 1:**
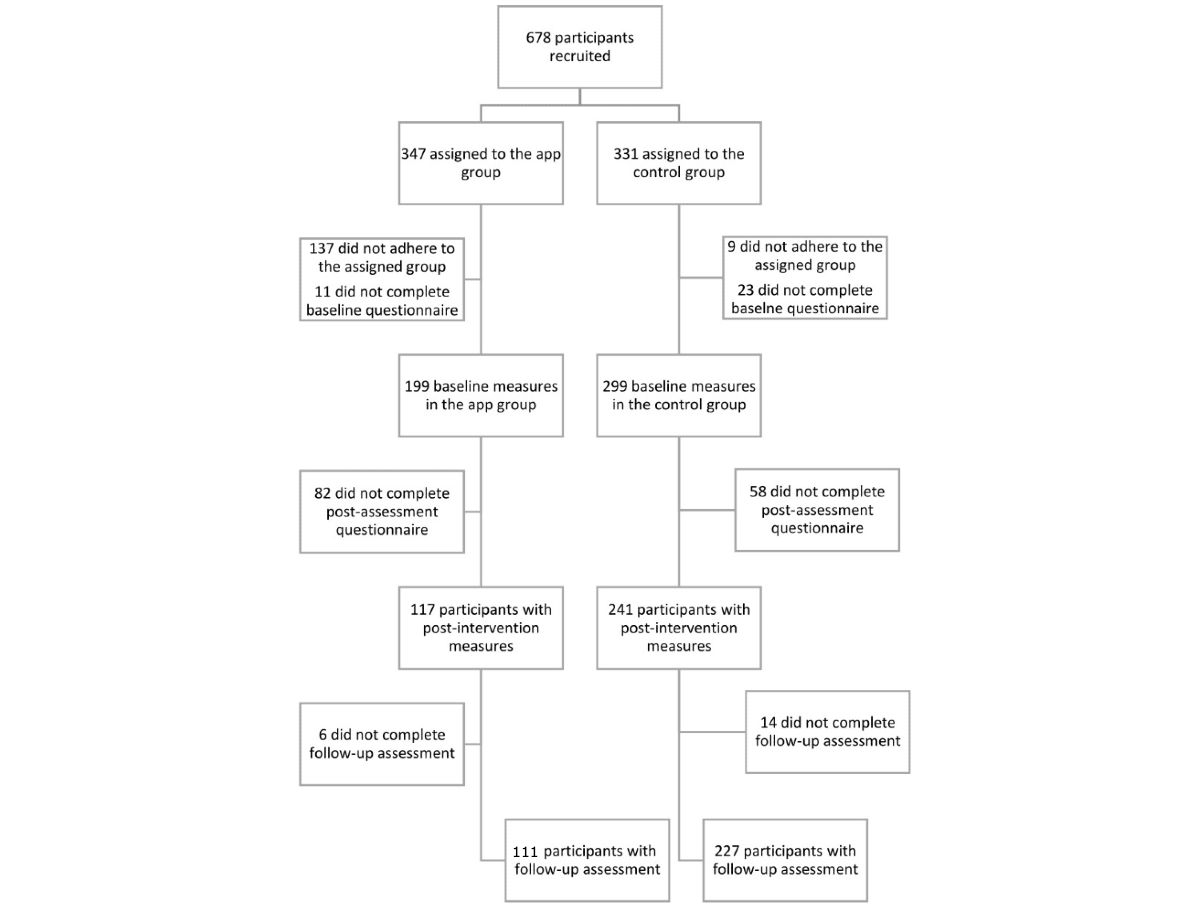
Participation flow diagram.

The recruitment of employees from the private and public sector took place in six different European businesses in Germany, England, and Northern Ireland. Potential participants were informed about trial participation through intraorganization communication channels such as emails, newsletters, intranet posts, or word of mouth, which varied by trial site. In total, 678 participants were recruited, of whom 621 completed the questionnaires at baseline, 483 completed the midintervention questionnaires, 396 completed the postintervention questionnaires, and 363 completed the 2-week follow-up questionnaires. Of the participants randomized into the app group (intervention group), 137 did not use the app at all and were therefore excluded from the analyses. Nine participants in the waitlist group downloaded the app before the end of the trial and were also excluded. Thus, the final sample consisted of 532 participants, with 210 in the intervention group and 322 in the waitlist control group (Figure 1). Of the participants in the intervention group who adhered to their group allocation, the mean number of sessions completed was 11.06 (SD 7.34, range 1-28). Moreover, they tracked a mean of 3.61 nights (SD 6.11, range 0-25). However, the majority of participants (n=111, 52.9%) did not track their sleep at all and 22 (10.5%) of them tracked their sleep only once.

### Kelaa Mental Resilience App

Details of the app-based intervention tool have been previously reported [29]. Briefly, the Kelaa Mental Resilience app aimed at increasing well-being and reducing stress of users in a workplace context, using techniques based on cognitive behavioral therapy (CBT) and mindfulness, among others. The app entails a tracking module that measures behavior, cognitions, and emotions, and an intervention module with psychoeducational content. Using these two modules, “Kelaa” was designed to implement lifestyle changes for its users.

**Figure 2:**
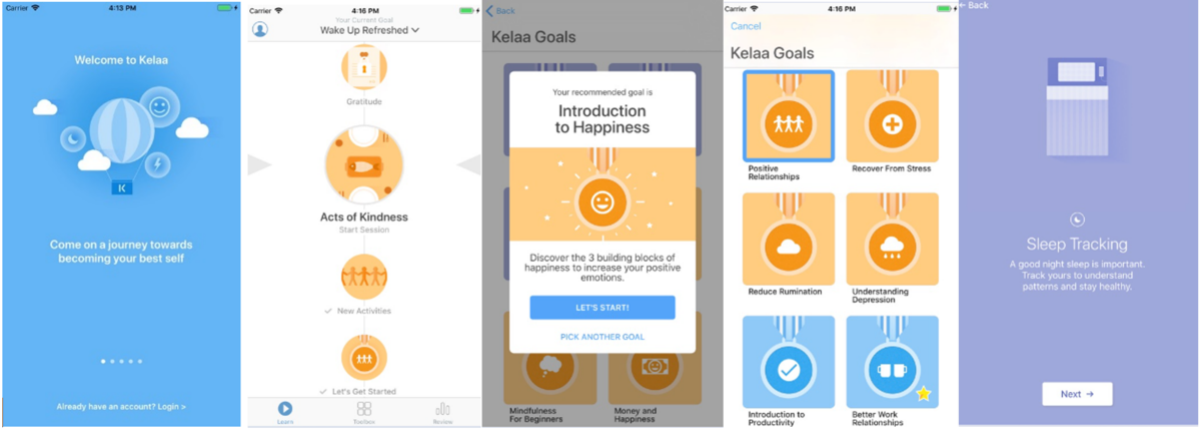
Screenshots of the Kelaa Mental Resilience app. Copyright Soma Analytics United Kingdom.

In the tracking module, through the use of scientifically validated questionnaires, the users of the app could track their stress levels, well-being, and resilience. Additionally, using inbuilt sensors (eg, accelerometer), they were also able to monitor their sleep quantity and quality. They were then given personalized feedback according to their questionnaire scores and sleep data. Within the intervention module, “Kelaa” provided content in different categories (“Kelaa Goals”), including stress recovery, happiness, or sleep. Within these weekly topics, users were provided with evidence-based interventions based on current research such as CBT, positive psychology, and mindfulness. All users had the opportunity to choose any topic that suited them best based on their outcomes resulting from the tracking module and personal interest. After choosing a goal, the user received six to seven “daily sessions” that were gradually unlocked, guiding them through their self-selected goals. Figure 2 displays some of the content provided by “Kelaa.”

### Measures

#### Productivity Measures

The impact of health problems on the ability to undertake regular activities and to work over the previous week was assessed by means of the Work Productivity and Activity Impairment Questionnaire: General Health V2.0 (WPAI:GH) [33].

#### Absenteeism

For absenteeism, the scores were calculated according to the following formula:

[Number of hours missed from work because of health problems/(Number of hours missed from work because of health problems+Number of hours worked)]×100

This resulted in a percentage of work time missed due to health ranging from 0 to 100, where 0 represents no impairment and 100 represents that the person was completely absent from work during the previous 7 days.

#### Presenteeism

Based on the answer to the question “During the past 7 days, how much did health problems affect your productivity while you were working?,” the proportion of impairment while working due to health was calculated. This measure multiplied by 100 was used as a measure of presenteeism. This yielded a score of 0 to 100, where 0 represents no presenteeism and 100 indicates the maximum amount of presenteeism.

#### Stress

The Copenhagen Psychosocial Questionnaire-Revised Version (COPSOQ II) [34] evaluates the levels of stress with two subscales: General Stress (4 items; eg, “How often have you been stressed?”) and Cognitive Stress (4 items; eg, “How often have you had problems concentrating?”). A 5-point Likert scale (1=not at all to 5=all the time) was used, where lower values indicate lower levels of stress.

### Ethical Considerations

Ethics approval was granted by an Independent Ethics Advisory Board (EAB) according to the European Commission Horizon 2020 Ethics Appraisal Procedure [35], which consisted of four independent ethics advisors from Germany and the United Kingdom. All members of the EAB evaluated the project as either “favorable opinion” or “favorable opinion with additional conditions.” Ethics approval was required and obtained as per applicable institutional and national guidelines and regulations. Data protection policies were strictly followed according to General Data Protection Regulation guidelines. Participation in the study was voluntary. All participants gave their informed consent in written form. The participants received no compensation for their participation.

### Statistical Analysis

Descriptive statistics of the variables of interest were calculated for all the participants and for the two groups (intervention group vs control group) separately. Wilcoxon rank-sum tests and *χ*^2^ tests were used to test for differences between the two groups on these measures.

To test the intervention effect on presenteeism and absenteeism, we used multilevel modeling for repeated measures with measurements (level 1) nested within subjects (level 2). Since the number of cases for each level would have been less than 30 [36], the “trial site” was not included as a third level in the analysis. Multilevel-measure ANOVAs are more robust with respect to missing data and underlying variance/covariance assumptions than repeated-measures ANOVAs [36]. In the models, the intercept and subject-specific treatment effect may randomly vary across subjects (random intercepts and slopes model). Two different multilevel models were calculated, with presenteeism and absenteeism as the respective outcomes. Time (T0, T1, T2, and T3), group (intervention vs control), and their interaction were included in the model as predictors. Gender and age were included as covariates. After determining whether there was a direct treatment effect, the effect of general and cognitive stress at baseline on the outcomes of interest was estimated. Holm-Bonferroni *P* value corrections were performed for these analyses. Analyses regarding the effect of the intervention on mental health variables were performed in the aforementioned previous study [29].

We constructed mediational models to determine the mediating role of general and cognitive stress in the association between groups (intervention vs control) and presenteeism and absenteeism [37]. Eight different mediation models were estimated by combining the outcome (presenteeism or absenteeism), mediator (general or cognitive stress), and time point (postintervention or follow-up). Models were adjusted for gender, age, baseline level of the dependent variable, and baseline level of the mediator. Bootstrap techniques with 5000 simulations were used [38,39]. β coefficients with *P* values for direct, indirect, and total effects and their 95% CIs were calculated. The direct effect refers to the effect of the independent variable on the outcome when the mediator is included in the model. The indirect effect represents the effect that the independent variable has on the dependent variable that is explained by this mediator [38,39]. A significant indirect effect indicates mediation in the analyses, irrespective of whether the total or direct effects are significant [40]. If the indirect effects result is significant, a partially standardized indirect effect will be estimated (*ab_ps_*) as a measure of the size of the effect [41,42]. This estimate calculates the expected decrease in SDs of the dependent variable for every 1-unit increase in the independent variable indirectly via the mediator.

Data were analyzed using the statistical programming language R [43] in combination with the *nlme* package [44] and the *mediate* package for the mediation analyses [45]. As both the mediator and the outcome model were linear models, the results obtained by the function are analogous to the usual linear structural equation method described by Baron and Kenny [45,46].

## Results

### Descriptive Analyses

A summary of the demographic data, stress scores, and presenteeism and absenteeism information of the participants at baseline is provided in Table 1. The mean age in the overall sample was 40.6 (SD 11.2) years, with 75.6% women. Comparisons between the intervention and control groups in terms of sociodemographics, cognitive and general stress, presenteeism, and absenteeism yielded nonsignificant results (all *P*>.05).

**Table 1:** Descriptive analyses for baseline measures by group.

Variable (range)			Total				App intervention group				Waitlist control group			Statistic^a^		*P* value
			Responses, n		Value		Responses, n		Value		Responses, n		Value			
Age (17-72 years), mean (SD)			485		40.62 (11.19)		198		39.95 (10.95)		287		41.08 (11.35)	30,168		.25
Cognitive stress (1-5), mean (SD)			496		2.61 (0.81)		199		2.59 (0.85)		297		2.63 (0.78)	31,010		.35
General stress (1-5), mean (SD)			498		3.01 (0.73)		199		3.00 (0.76)		299		3.01 (0.73)	30,105		.82
Presenteeism (0-100), mean (SD)			464		20.58 (21.86)		186		21.78 (22.78)		278		19.78 (21.23)	25,038		.56
Absenteeism (0-100), mean (SD)			465		4.08 (13.47)		186		3.50 (11.54)		279		4.46 (14.62)	25,736		.83
**Gender, n (%)**			488				198				290			0.0022 (*df*=1)		.96
	Female			369 (75.6)				149 (75.3)				220 (75.9)				
	Male			119 (24.4)				49 (24.8)				70 (24.1)				
**Language, n (%)**			489				199				290			0.1471 (*df*=1)		.70
	Native			365 (74.6)		214		214 (73.8)		151		151 (75.9)				
	Nonnative			124 (25.4)		76		76 (26.2)		48		48 (24.1)				

^a^Wilcoxon rank-sum statistic for continuous variables and *χ*^2^ statistic for categorical variables.

### Effect of the Intervention on Work-Related Outcomes

In contrast to our first hypothesis, there was no direct effect of the intervention on either absenteeism or presenteeism. Time significantly predicted presenteeism, but not absenteeism. The time-by-intervention interaction was not significant in the models for presenteeism or absenteeism. Thus, further analyses were performed without adding the time-by-group interaction into the models.

Supporting our second hypothesis, levels of presenteeism were found to be higher for participants who had a higher level of general stress and a higher level of cognitive stress (see Table 2). For absenteeism, only cognitive stress was found to be a significant predictor, where absenteeism was found to be higher for participants with higher cognitive stress scores. General stress did not predict absenteeism (Table 2).

**Table 2:** Adjusted multilevel models for repeated measures for the effect of general and cognitive stress on presenteeism and absenteeism (N=484).

Predictors	Presenteeism		Absenteeism	
	Estimate, β (95% CI)	*P* value^a^	Estimate, β (95% CI)	*P* value^a^
Intercept	–14.00 (–22.72 to –5.27)	.007	–.30 (–5.72 to 5.12)	>.99
Age	–.02 (–.17 to –.12)	>.99	–.05 (–.13 to .04)	>.99
Gender	–.41 (–4.19 to –2.63)	>.99	1.26 (–.98 to 3.50)	>.99
Time	1.70 (.76 to 2.63)	.002	.89 (.13 to 1.64)	.13
Intervention group	2.47 (–.89 to 5.83)	.45	–.14 (–2.14 to 1.85)	>.99
Cognitive stress	8.79 (6.74 to 10.84)	<.001	2.01 (.62 to 3.39)	.03
General stress	4.50 (2.46 to 6.53)	<.001	–.08 (–1.51 to 1.34)	>.99

^a^*P* values were adjusted using the Holm-Bonferroni correction.

### Mediation Analyses

Four mediational models were calculated separately for each outcome and each mediator. Partially supporting our third hypothesis, general stress significantly mediated the relationship between the intervention group and presenteeism at T2 (postintervention, *P*=.005). The partially standardized indirect effect (*ab_ps_*) was –0.051; as such, presenteeism decreased by –0.051 when pertaining to the intervention group as compared to the control group indirectly via general stress. However, this was not the case for absenteeism (*P*=.92) (see Table 3). The direct and total effects of the intervention on presenteeism and absenteeism were nonsignificant (all *P*>.05). Cognitive stress, in turn, significantly mediated the relationship between the intervention and presenteeism (*ab_ps_*=–0.098, *P*<.001) as well as absenteeism (*ab_ps_*=–0.053, *P*=.02). Again, the direct and total effects of the intervention group on presenteeism and absenteeism were nonsignificant (all *P*>.05).

Mediation analyses were repeated for the outcomes at the 2-week follow-up (Table 4). While general stress did not significantly mediate the relationship between the intervention effects and the outcome measures (all *P*>.05), cognitive stress mediated the relationship between the intervention effects and presenteeism (*ab_ps_*=–0.064, *P*=.04), but not the relationship with absenteeism (*P*=.36). In addition, the direct and total effects of the intervention group on presenteeism and absenteeism were nonsignificant (all *P*>.05).

**Table 3:** Mediation analyses with group as the independent variable at postintervention.^a^

Outcome and mediator			Coefficient, β (95% CI)	*P* value
**Presenteeism (n=333)**				
	**General stress as mediator**			
		Total effect	1.10 (–4.33 to 6.88)	.70
		Direct effect	2.46 (–2.85 to 8.27)	.39
		Indirect effect	–1.36 (–2.91 to –0.28)	.005
	**Cognitive stress as mediator**			
		Total effect	1.15 (–4.34 to 6.85)	.70
		Direct effect	3.78 (–1.54 to 9.44)	.18
		Indirect effect	–2.64 (–4.55 to –1.08)	<.001
**Absenteeism (n=288)**				
	**General stress as mediator**			
		Total effect	–.74 (–4.39 to 3.35)	.70
		Direct effect	–.77 (–4.47 to 3.04)	.66
		Indirect effect	.04 (–.61 to 1.01)	.92
	**Cognitive stress as mediator**			
		Total effect	–.84 (–4.50 to 3.28)	.66
		Direct effect	.05 (–3.26 to 3.85)	.99
		Indirect effect	–.89 (–2.00 to –.09)	.02

^a^Model adjusted for gender, age, baseline measure of mediator, and baseline measure of outcome.

**Table 4:** Mediation analyses with group as the independent variable at 2-week follow-up.^a^

Outcome and mediator				Coefficient, β (95% CI)		*P* value
**Presenteeism**						
	**General stress as mediator (n=310)**					
		Total effect	1.50 (–3.82 to 7.24)		.58	
		Direct effect	2.28 (–2.90 to 7.69)		.40	
		Indirect effect	–.78 (–2.19 to .51)		.25	
	**Cognitive stress as mediator (n=312)**					
		Total effect	1.60 (–3.34 to 7.38)		.53	
		Direct effect	3.24 (–1.56 to 8.68)		.78	
		Indirect effect	–1.63 (–3.54 to .02)		.04	
**Absenteeism**						
	**General stress as mediator (n=260)**					
		Total effect	.98 (–4.05 to 6.57)		.74	
		Direct effect	1.11 (–3.89 to 6.73)		.70	
		Indirect effect	–.12 (–.88 to .42)		.72	
	**Cognitive stress as mediator (n=263)**					
		Total effect	1.07 (–4.03 to 6.58)		.70	
		Direct effect	1.35 (–3.69 to 6.84)		.62	
		Indirect effect	–.28 (–1.51 to .25)		.36	

^a^Model adjusted for gender, age, baseline measure of mediator, and baseline measure of outcome.

## Discussion

### Principal Findings

The Kelaa Mental Resilience app, a mobile-based intervention, has previously been shown to be effective in reducing stress in workers [29]. In this study, we aimed to determine if this intervention was also effective in reducing levels of presenteeism and absenteeism, and to analyze the mediating role of stress on these measures among workers. Our findings showed no direct effect of the intervention on the levels of presenteeism and absenteeism. However, as the intervention was found to decrease stress [29], and we found stress to be related to presenteeism and absenteeism scores, we tested whether there was an indirect intervention effect on presenteeism and absenteeism through improvement of stress outcomes. Mediation analyses were performed separately for both cognitive and general stress as mediators and for both presenteeism and absenteeism as outcomes at postintervention (T2) and at follow-up (T3). We found an indirect effect of the intervention on presenteeism and absenteeism through both cognitive and general stress. This suggests that while the intervention itself does not directly impact presenteeism and absenteeism, it may indirectly influence work outcomes through the impact on stress levels. However, the effects seem to only persist right after participants had finished the intervention (T2), except for the mediation effect of cognitive stress on presenteeism, which remained significant at T3.

In contrast to our findings, some previous studies reported significant effects of digital workplace interventions on presenteeism and absenteeism [13,14]. However, the indirect effect of cognitive and general stress on both presenteeism and absenteeism found in this study is in line with the JDR model [21,22], which states that when demands exceed resources, individuals tend to experience higher stress, leading to a decrease in energy and exhaustion. In contrast, when workers’ resources are greater than the demands that work places on them, they undergo a motivational process, which may translate into an increase in work engagement and positive outcomes [23-25]. As such, in this study, individuals who experienced more stress were also more prone to report higher absenteeism and presenteeism scores. This finding is also in line with previous studies that found a relationship between stress and productivity [16-18]. The mediated effect of the intervention on productivity through stress aligns with the assumption that job control is closely related to cognitive stress [20]. In the mediation analyses, no direct or total effects were found in any of the models. This is possible, as an indirect effect can exist in the absence of total effects since a total effect is the sum of many different paths of influence and not all of them are represented by the model [38]. This points toward the possibility of another “hidden” mediator with opposing signs as compared to the already identified mediators cognitive and general stress that is potentially responsible for the nonexistent direct intervention effect on presenteeism and absenteeism [47]. Further research is needed to explore the different pathways through which interventions might affect productivity scores.

### Strengths, Limitations, and Practical Implications

This research has several strengths. The fact that the study was conducted in different work environments, disciplines, and countries allowed the results to represent different working conditions. Furthermore, the Kelaa Mental Resilience app is grounded both in theory (ie, the JDR model [21]) and entails a variety of evidence-based interventions targeting specific challenges that an employee might face in a workplace context.

Some limitations also need to be considered when interpreting our results. First, the sample is not representative of the general working population as there was an overrepresentation of female participants and the educational level of the participants was high. Thus, the results might not be generalized to other workplace settings. Second, the scales used to assess our outcomes may not have been sensitive enough to capture smaller changes that occurred. Additionally, as a technical constraint, the range of answers sometimes exceeded the size of the mobile phone screen and would have required scrolling to see the full scale, which may have resulted in an overrepresentation of the scores displayed on the screen. Third, the intervention was personalized according to the workers’ profiles, making it difficult to identify which elements contributed to the effects found. For such analyses, more data points and a larger data set would be needed. Fourth, participants were not blinded to the intervention allocation, which may have increased the bias. Fifth, attrition during the study was high; 47.2% of the participants had dropped out at the postintervention time pint (T2), while 50.1% had dropped out at follow-up (T3). Such high attrition adds bias to the results of the study, as it might have resulted in a self-selection mechanism and therefore limit the conclusions drawn from the study, consequently affecting the internal validity of the results. Related to this, the Kelaa Mental Resilience app might not have been suitable for everyone, again resulting in a self-selection mechanism and high attrition rates.

A broader scope of the app content could result in a more applicable intervention for a larger variety of users. High attrition rates can also suggest that better communication strategies are needed in the trial sites to increase users’ engagement. Generally, understanding the needs of the target population may lead to better outcomes by providing them with an intervention fitting their needs [48]. Along with posing a problem on the internal validity of the study, attrition also causes low actual use of the app by the participants who remained in the study, leading to compliance bias. Intervention-compliant participants may differ from noncompliant participants in ways that influence the outcomes of the study. Moreover, when using per-protocol analyses, the results are not representative of the real-life situation and lead to exaggerated treatment effects for which the conclusions should be interpreted cautiously. Finally, although the naturalistic nature of the study granted external validity, the differences in workplace context might have introduced a range of factors that could not be controlled for, resulting in low internal validity.

### Conclusion

Overall, our findings suggest that the effects of a mobile mental health intervention on stress levels might have a positive effect on presenteeism and absenteeism in workers. However, such effects do not seem to be persistent 2 weeks after finishing using the intervention, except in the case of the impact of the interventions via cognitive stress on presenteeism. While further studies are needed to replicate our findings and examine further pathways through which mobile mental health interventions might influence workers’ productivity, our results add to the sparse research on the mechanisms of preventative e-mental health interventions in the workplace regarding productivity outcomes. This study adds evidence of the impact of the intervention on productivity measures. Future interventional studies focusing on mental health in the workplace context should consider both theory and evidence when further developing interventions. Future studies should include a more representative sample and a longer follow-up to further disentangle the effects of e-mental health in occupational settings.

## Appendix

Multimedia Appendix 1 CONSORT EHEALTH Checklist (V 1.6.1).

## Abbreviations

CBT: cognitive behavioral therapy
COPSOQ II: Copenhagen Psychosocial Questionnaire-Revised Version
EAB: Ethics Advisory Board
e-mental health: electronic mental health
JDR: Job Demands-Resources model
RCT: randomized controlled trial
WPAI:GH: Work Productivity and Activity Impairment Questionnaire: General Health V2.0

## Data Availability

The data sets generated during and/or analyzed during the current study are available from author SW on reasonable request.
